# (5,15-Dianthracen-9-yl-10,20-dihexyl­porphyrinato)nickel(II): a planar nickel(II) porphyrin

**DOI:** 10.1107/S1600536810021434

**Published:** 2010-06-16

**Authors:** Mathias O. Senge, Mia Davis

**Affiliations:** a SFI Tetrapyrrole Laboratory, School of Chemistry, Trinity College Dublin, Dublin 2, Ireland

## Abstract

The title compound, [Ni(C_60_H_52_N_4_)], is an example of a *meso* tetra­substituted nickel(II) porphyrin with both *meso* aryl and alkyl residues. The mol­ecule exhibits a planar macrocycle with an average deviation of the 24 macrocycle atoms from their least-squares plane (Δ24) of 0.01 Å and an average Ni—N bond length of 1.960 (2) Å. The Ni^II^ atom lies on a center of inversion. The structure presents a rare example for a planar nickel(II) porphyrin, as *meso*-substituted nickel(II) porphyrins with either only *meso*-aryl or with *meso*-alkyl residues typically exhibit a ruffled conformation.

## Related literature

For the conformation of porphyrins, see: Senge (2006 ▶). For porphyrins with mixed *meso* substituents, see: Senge *et al.* (2010 ▶). For Ni(II) porphyrin structures, see: Fleischer *et al.* (1964 ▶); Gallucci *et al.* (1982 ▶); Hoard (1973 ▶); Lee & Scheidt (1987 ▶); Senge *et al.* (1999 ▶, 2000 ▶) and Runge *et al.* (1999 ▶). For anthracenyl porphyrins see: Volz & Schäffer (1985 ▶); Davis *et al.* (2008 ▶); Sooambar *et al.* (2009 ▶). For the handling of the crystals, see: Hope (1994 ▶).
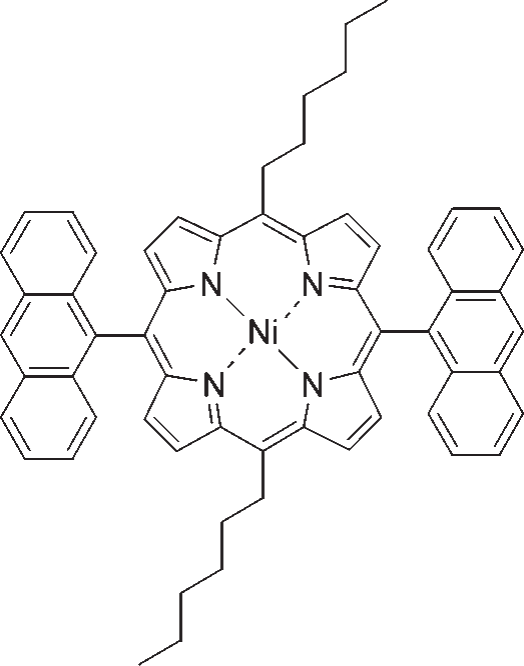


## Experimental

### 

#### Crystal data

[Ni(C_60_H_52_N_4_)]
*M* *_r_* = 887.77
Triclinic, 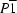

*a* = 7.797 (3) Å
*b* = 9.387 (3) Å
*c* = 15.285 (5) Å
α = 97.246 (6)°
β = 91.222 (4)°
γ = 91.402 (6)°
*V* = 1109.1 (7) Å^3^
*Z* = 1
Mo *K*α radiation
μ = 0.48 mm^−1^
*T* = 118 K
0.50 × 0.20 × 0.05 mm

#### Data collection

Rigaku Saturn724 diffractometer
17330 measured reflections
3875 independent reflections
3233 reflections with *I* > 2σ(*I*)
*R*_int_ = 0.070

#### Refinement

*R*[*F* ^2^ > 2σ(*F* ^2^)] = 0.039
*wR*(*F* ^2^) = 0.087
*S* = 1.00
3875 reflections
296 parameters
H-atom parameters constrained
Δρ_max_ = 0.37 e Å^−3^
Δρ_min_ = −0.40 e Å^−3^

### 

Data collection: *CrystalClear* (Rigaku, 2008 ▶); cell refinement: *CrystalClear*; data reduction: *CrystalClear*; program(s) used to solve structure: *SHELXS97* (Sheldrick, 2008 ▶); program(s) used to refine structure: *SHELXL97* (Sheldrick, 2008 ▶); molecular graphics: *SHELXTL* (Sheldrick, 2008 ▶); software used to prepare material for publication: *SHELXL97*.

## Supplementary Material

Crystal structure: contains datablocks I, global. DOI: 10.1107/S1600536810021434/ng2783sup1.cif

Structure factors: contains datablocks I. DOI: 10.1107/S1600536810021434/ng2783Isup2.hkl

Additional supplementary materials:  crystallographic information; 3D view; checkCIF report

## Figures and Tables

**Table 1:** Selected bond lengths (Å)

Ni—N22	1.9570 (17)
Ni—N21	1.9632 (17)

**Fig. 1.**
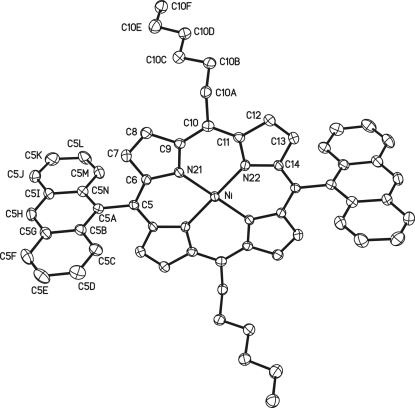
: View of the molecular structure of I in the crystals. Thermal ellipsoids are drawn for 50% occupancy.

## supplementary crystallographic information

### Comment

In continuation of studies on the conformational flexibility of porphyrins (Senge, 2006) the structure of the title compound was determined as an example for a *meso* substituted porphyrin with both *meso* alkyl and *meso* aryl subsitutents (Senge *et al.*, 2010) and in relation to current synthetic studies on anthracenyl porphyrins (Volz & Schäffer, 1985; Davis *et al.*, 2008; Sooambar *et al.*, 2009).

The structure of (I), is shown in Fig. 1. The molecule exhibits a completely planar macrocycle with an average deviation of the 24 macrocycle atoms from their least-squares-plane (Δ24) of 0.01 Å and an average Ni—N bond length of 1.960 (2) Å. All geometrical parameters are typical for a planar nickel(II) porphyrin (Senge *et al.*, 2000). No individual macrocycle atom was displaced more then 0.015 Å from the mean plane. Likewise, differences in bond angles and lenghts between the *meso* aryl and *meso* alkyl quadrants are minimal. The anthracenyl residues are almost orthogonal to the plane of the four nitrogen atoms (96.2°) similarly to the situation found in related zinc(II) systems with *meso* aryl residues (Sooambar *et al.*, 2009). In the crystal packing there are no close contacts (not shown). The anthracene residues prevent π-stacking of the porphyrins and the hexyl side chains are oriented between neighboring anthracenyl substituents and hinder π-stacking as well.

The structure presents a rare example for a planar nickel(II) porphyrin. Typically, *meso* substituted nickel(II) porphyrins with only *meso* aryl residues (Fleischer *et al.*, 1964; Hoard, 1973; Lee & Scheidt, 1987) and those with *meso* alkyl residues (Senge *et al.*, 1999; Runge *et al.*, 1999) exhibit a ruffled conformation. Only (5,10,15,20-tetramethylporphyrinato)nickel(II) exbihits an almost planar conformation as well (Gallucci *et al.*, 1982).

### Experimental

The compound was prepared *via* metallation of the respective free base porphyrin and crystallized *via* liquid diffusion of methanol into a solution of the porphyrin in methylene chloride. Crystals were handled as described by Hope (1994).

### Refinement

Hydrogen atoms were located in difference maps and refined using a standard riding model.

### Crystal data

**Table:**

[Ni(C_60_H_52_N_4_)]	*Z* = 1
*M**_r_* = 887.77	*F*(000) = 468
Triclinic, *P*1	*D*_x_ = 1.329 Mg m^−^^3^
Hall symbol: -P 1	Melting point: n/d K
*a* = 7.797 (3) Å	Mo *K*α radiation, λ = 0.71075 Å
*b* = 9.387 (3) Å	Cell parameters from 3864 reflections
*c* = 15.285 (5) Å	θ = 2.4–31.2°
α = 97.246 (6)°	µ = 0.48 mm^−^^1^
β = 91.222 (4)°	*T* = 118 K
γ = 91.402 (6)°	Prism, red
*V* = 1109.1 (7) Å^3^	0.50 × 0.20 × 0.05 mm

### Data collection

**Table:**

Rigaku Saturn724 diffractometer	3233 reflections with *I* > 2σ(*I*)
Radiation source: Sealed Tube	*R*_int_ = 0.070
Graphite Monochromator	θ_max_ = 25.0°, θ_min_ = 3.0°
Detector resolution: 28.5714 pixels mm^-1^	*h* = −9→9
dtprofit.ref scans	*k* = −11→11
17330 measured reflections	*l* = −18→18
3875 independent reflections	

### Refinement

**Table:**

Refinement on *F*^2^	Primary atom site location: structure-invariant direct methods
Least-squares matrix: full	Secondary atom site location: difference Fourier map
*R*[*F*^2^ > 2σ(*F*^2^)] = 0.039	Hydrogen site location: inferred from neighbouring sites
*wR*(*F*^2^) = 0.087	H-atom parameters constrained
*S* = 1.00	*w* = 1/[σ^2^(*F*_o_^2^) + (0.0371*P*)^2^] where *P* = (*F*_o_^2^ + 2*F*_c_^2^)/3
3875 reflections	(Δ/σ)_max_ < 0.001
296 parameters	Δρ_max_ = 0.37 e Å^−^^3^
0 restraints	Δρ_min_ = −0.40 e Å^−^^3^
0 constraints	

### Special details

**Table:**

Geometry. All e.s.d.'s (except the e.s.d. in the dihedral angle between two l.s. planes) are estimated using the full covariance matrix. The cell e.s.d.'s are taken into account individually in the estimation of e.s.d.'s in distances, angles and torsion angles; correlations between e.s.d.'s in cell parameters are only used when they are defined by crystal symmetry. An approximate (isotropic) treatment of cell e.s.d.'s is used for estimating e.s.d.'s involving l.s. planes.
Refinement. Refinement of *F*^2^ against ALL reflections. The weighted *R*-factor *wR* and goodness of fit *S* are based on *F*^2^, conventional *R*-factors *R* are based on *F*, with *F* set to zero for negative *F*^2^. The threshold expression of *F*^2^ > σ(*F*^2^) is used only for calculating *R*-factors(gt) *etc*. and is not relevant to the choice of reflections for refinement. *R*-factors based on *F*^2^ are statistically about twice as large as those based on *F*, and *R*- factors based on ALL data will be even larger.

### Fractional atomic coordinates and isotropic or equivalent isotropic displacement parameters (Å^2^)

**Table:**

	*x*	*y*	*z*	*U*_iso_*/*U*_eq_	
Ni	0.0000	0.5000	0.0000	0.01798 (12)	
N21	0.1442 (2)	0.47152 (16)	0.10290 (10)	0.0185 (4)	
N22	0.1822 (2)	0.62389 (16)	−0.03788 (10)	0.0192 (4)	
C5	−0.0499 (3)	0.31349 (19)	0.17545 (13)	0.0185 (4)	
C6	0.1022 (3)	0.3901 (2)	0.16960 (13)	0.0198 (5)	
C7	0.2375 (3)	0.3969 (2)	0.23403 (13)	0.0247 (5)	
H7A	0.2383	0.3500	0.2856	0.030*	
C8	0.3643 (3)	0.4817 (2)	0.20886 (14)	0.0243 (5)	
H8A	0.4707	0.5059	0.2391	0.029*	
C9	0.3069 (3)	0.5284 (2)	0.12716 (13)	0.0194 (5)	
C10	0.4031 (3)	0.6194 (2)	0.08102 (13)	0.0204 (5)	
C11	0.3407 (3)	0.6617 (2)	0.00254 (13)	0.0197 (5)	
C12	0.4362 (3)	0.7542 (2)	−0.04785 (14)	0.0244 (5)	
H12A	0.5476	0.7949	−0.0337	0.029*	
C13	0.3384 (3)	0.7725 (2)	−0.11872 (14)	0.0248 (5)	
H13A	0.3677	0.8283	−0.1642	0.030*	
C14	0.1820 (3)	0.69232 (19)	−0.11298 (13)	0.0190 (5)	
C5A	−0.0738 (3)	0.2335 (2)	0.25355 (13)	0.0205 (5)	
C5B	−0.0381 (3)	0.0855 (2)	0.24777 (14)	0.0230 (5)	
C5C	0.0207 (3)	0.0055 (2)	0.16833 (15)	0.0263 (5)	
H5CA	0.0405	0.0534	0.1181	0.032*	
C5D	0.0488 (3)	−0.1376 (2)	0.16363 (16)	0.0334 (6)	
H5DA	0.0880	−0.1884	0.1104	0.040*	
C5E	0.0197 (3)	−0.2119 (2)	0.23800 (17)	0.0360 (6)	
H5EA	0.0382	−0.3122	0.2338	0.043*	
C5F	−0.0339 (3)	−0.1403 (2)	0.31441 (16)	0.0321 (6)	
H5FA	−0.0517	−0.1913	0.3635	0.038*	
C5G	−0.0645 (3)	0.0104 (2)	0.32312 (14)	0.0255 (5)	
C5H	−0.1190 (3)	0.0864 (2)	0.40177 (14)	0.0276 (5)	
H5HA	−0.1338	0.0367	0.4516	0.033*	
C5I	−0.1523 (3)	0.2321 (2)	0.40970 (13)	0.0230 (5)	
C5J	−0.2098 (3)	0.3101 (2)	0.48906 (14)	0.0318 (6)	
H5JA	−0.2196	0.2628	0.5402	0.038*	
C5K	−0.2508 (3)	0.4502 (2)	0.49355 (15)	0.0393 (6)	
H5KA	−0.2877	0.5003	0.5475	0.047*	
C5L	−0.2383 (3)	0.5230 (2)	0.41624 (15)	0.0347 (6)	
H5LA	−0.2719	0.6200	0.4186	0.042*	
C5M	−0.1797 (3)	0.4549 (2)	0.34139 (15)	0.0298 (5)	
H5MA	−0.1677	0.5061	0.2920	0.036*	
C5N	−0.1344 (3)	0.3070 (2)	0.33320 (13)	0.0230 (5)	
C10A	0.5771 (3)	0.6803 (2)	0.11646 (13)	0.0235 (5)	
H10A	0.6577	0.6781	0.0671	0.028*	
H10B	0.6232	0.6189	0.1591	0.028*	
C10B	0.5667 (3)	0.8359 (2)	0.16236 (13)	0.0265 (5)	
H10C	0.6835	0.8805	0.1670	0.032*	
H10D	0.4955	0.8913	0.1250	0.032*	
C10C	0.4915 (3)	0.8477 (2)	0.25444 (14)	0.0279 (5)	
H10E	0.5718	0.8051	0.2943	0.033*	
H10F	0.3823	0.7909	0.2514	0.033*	
C10D	0.4574 (3)	1.0030 (2)	0.29382 (14)	0.0287 (5)	
H10G	0.5637	1.0622	0.2916	0.034*	
H10H	0.3681	1.0425	0.2574	0.034*	
C10E	0.4000 (3)	1.0138 (2)	0.38747 (15)	0.0356 (6)	
H10I	0.4938	0.9824	0.4246	0.043*	
H10J	0.3004	0.9473	0.3904	0.043*	
C10F	0.3500 (3)	1.1641 (2)	0.42523 (14)	0.0316 (6)	
H10K	0.3292	1.1666	0.4884	0.047*	
H10L	0.2453	1.1900	0.3950	0.047*	
H10M	0.4430	1.2326	0.4166	0.047*	

### Atomic displacement parameters (Å^2^)

**Table:**

	*U*^11^	*U*^22^	*U*^33^	*U*^12^	*U*^13^	*U*^23^
Ni	0.0227 (2)	0.0162 (2)	0.0154 (2)	−0.00037 (16)	0.00200 (15)	0.00351 (15)
N21	0.0227 (10)	0.0156 (8)	0.0174 (9)	−0.0013 (7)	0.0034 (7)	0.0021 (7)
N22	0.0240 (10)	0.0184 (9)	0.0155 (9)	0.0016 (8)	0.0008 (7)	0.0030 (7)
C5	0.0243 (12)	0.0137 (10)	0.0171 (10)	−0.0003 (9)	0.0026 (9)	0.0004 (8)
C6	0.0279 (13)	0.0145 (10)	0.0170 (11)	0.0015 (9)	0.0004 (9)	0.0020 (8)
C7	0.0307 (13)	0.0231 (11)	0.0212 (11)	−0.0003 (10)	−0.0011 (10)	0.0069 (9)
C8	0.0244 (12)	0.0235 (11)	0.0252 (12)	−0.0005 (9)	−0.0043 (9)	0.0048 (9)
C9	0.0219 (12)	0.0161 (10)	0.0198 (11)	0.0005 (9)	0.0000 (9)	0.0005 (9)
C10	0.0215 (12)	0.0177 (10)	0.0211 (11)	0.0032 (9)	0.0025 (9)	−0.0015 (9)
C11	0.0208 (12)	0.0171 (10)	0.0204 (11)	−0.0007 (9)	0.0021 (9)	−0.0005 (9)
C12	0.0243 (12)	0.0245 (11)	0.0246 (12)	−0.0043 (9)	0.0021 (9)	0.0047 (9)
C13	0.0282 (13)	0.0248 (11)	0.0221 (11)	−0.0045 (10)	0.0024 (9)	0.0061 (9)
C14	0.0249 (12)	0.0138 (10)	0.0184 (10)	−0.0005 (9)	0.0027 (9)	0.0028 (8)
C5A	0.0206 (12)	0.0199 (11)	0.0209 (11)	−0.0033 (9)	−0.0013 (9)	0.0036 (9)
C5B	0.0215 (12)	0.0224 (11)	0.0254 (12)	−0.0028 (9)	−0.0011 (9)	0.0050 (9)
C5C	0.0235 (12)	0.0243 (12)	0.0312 (13)	−0.0006 (10)	0.0029 (10)	0.0029 (10)
C5D	0.0297 (14)	0.0295 (13)	0.0400 (14)	0.0025 (11)	0.0049 (11)	0.0001 (11)
C5E	0.0338 (15)	0.0204 (12)	0.0548 (17)	0.0030 (10)	0.0046 (12)	0.0073 (11)
C5F	0.0275 (14)	0.0275 (12)	0.0445 (15)	0.0023 (10)	0.0012 (11)	0.0166 (11)
C5G	0.0227 (12)	0.0242 (12)	0.0308 (12)	−0.0018 (9)	−0.0011 (10)	0.0088 (10)
C5H	0.0259 (13)	0.0333 (13)	0.0259 (12)	−0.0043 (10)	−0.0008 (10)	0.0135 (10)
C5I	0.0234 (12)	0.0244 (11)	0.0218 (11)	−0.0032 (9)	0.0020 (9)	0.0056 (9)
C5J	0.0353 (14)	0.0375 (14)	0.0232 (12)	−0.0075 (11)	0.0003 (10)	0.0075 (10)
C5K	0.0509 (17)	0.0376 (14)	0.0268 (13)	−0.0036 (12)	0.0092 (12)	−0.0069 (11)
C5L	0.0490 (17)	0.0188 (11)	0.0359 (14)	0.0024 (11)	0.0132 (12)	−0.0006 (10)
C5M	0.0370 (14)	0.0257 (12)	0.0282 (13)	−0.0016 (10)	0.0020 (10)	0.0090 (10)
C5N	0.0238 (12)	0.0220 (11)	0.0234 (11)	−0.0015 (9)	0.0003 (9)	0.0035 (9)
C10A	0.0212 (12)	0.0290 (12)	0.0209 (11)	−0.0013 (9)	0.0002 (9)	0.0059 (9)
C10B	0.0222 (12)	0.0306 (12)	0.0262 (12)	−0.0058 (10)	−0.0007 (10)	0.0035 (10)
C10C	0.0286 (13)	0.0284 (12)	0.0269 (12)	−0.0013 (10)	−0.0015 (10)	0.0048 (10)
C10D	0.0265 (13)	0.0316 (12)	0.0282 (12)	−0.0031 (10)	−0.0030 (10)	0.0057 (10)
C10E	0.0428 (16)	0.0354 (13)	0.0285 (13)	0.0020 (11)	0.0001 (11)	0.0033 (11)
C10F	0.0368 (15)	0.0288 (12)	0.0289 (13)	0.0039 (11)	0.0004 (11)	0.0019 (10)

### Geometric parameters (Å, °)

**Table:**

Ni—N22	1.9570 (17)	C5E—H5EA	0.9500
Ni—N22^i^	1.9570 (17)	C5F—C5G	1.430 (3)
Ni—N21^i^	1.9632 (17)	C5F—H5FA	0.9500
Ni—N21	1.9632 (17)	C5G—C5H	1.399 (3)
N21—C6	1.389 (2)	C5H—C5I	1.389 (3)
N21—C9	1.389 (3)	C5H—H5HA	0.9500
N22—C14	1.384 (2)	C5I—C5J	1.423 (3)
N22—C11	1.388 (3)	C5I—C5N	1.446 (3)
C5—C6	1.382 (3)	C5J—C5K	1.354 (3)
C5—C14^i^	1.385 (3)	C5J—H5JA	0.9500
C5—C5A	1.501 (3)	C5K—C5L	1.442 (3)
C6—C7	1.423 (3)	C5K—H5KA	0.9500
C7—C8	1.346 (3)	C5L—C5M	1.333 (3)
C7—H7A	0.9500	C5L—H5LA	0.9500
C8—C9	1.441 (3)	C5M—C5N	1.433 (3)
C8—H8A	0.9500	C5M—H5MA	0.9500
C9—C10	1.390 (3)	C10A—C10B	1.544 (3)
C10—C11	1.392 (3)	C10A—H10A	0.9900
C10—C10A	1.521 (3)	C10A—H10B	0.9900
C11—C12	1.436 (3)	C10B—C10C	1.529 (3)
C12—C13	1.343 (3)	C10B—H10C	0.9900
C12—H12A	0.9500	C10B—H10D	0.9900
C13—C14	1.427 (3)	C10C—C10D	1.538 (3)
C13—H13A	0.9500	C10C—H10E	0.9900
C14—C5^i^	1.385 (3)	C10C—H10F	0.9900
C5A—C5B	1.416 (3)	C10D—C10E	1.501 (3)
C5A—C5N	1.417 (3)	C10D—H10G	0.9900
C5B—C5C	1.435 (3)	C10D—H10H	0.9900
C5B—C5G	1.440 (3)	C10E—C10F	1.518 (3)
C5C—C5D	1.359 (3)	C10E—H10I	0.9900
C5C—H5CA	0.9500	C10E—H10J	0.9900
C5D—C5E	1.426 (3)	C10F—H10K	0.9800
C5D—H5DA	0.9500	C10F—H10L	0.9800
C5E—C5F	1.352 (3)	C10F—H10M	0.9800
			
N22—Ni—N22^i^	180.00 (8)	C5H—C5G—C5F	122.4 (2)
N22—Ni—N21^i^	91.02 (7)	C5H—C5G—C5B	119.23 (19)
N22^i^—Ni—N21^i^	88.98 (7)	C5F—C5G—C5B	118.4 (2)
N22—Ni—N21	88.98 (7)	C5I—C5H—C5G	122.40 (19)
N22^i^—Ni—N21	91.02 (7)	C5I—C5H—H5HA	118.8
N21^i^—Ni—N21	180.00 (9)	C5G—C5H—H5HA	118.8
C6—N21—C9	104.27 (16)	C5H—C5I—C5J	122.86 (19)
C6—N21—Ni	126.74 (14)	C5H—C5I—C5N	118.78 (19)
C9—N21—Ni	128.98 (13)	C5J—C5I—C5N	118.31 (19)
C14—N22—C11	104.18 (16)	C5K—C5J—C5I	121.6 (2)
C14—N22—Ni	127.19 (14)	C5K—C5J—H5JA	119.2
C11—N22—Ni	128.63 (13)	C5I—C5J—H5JA	119.2
C6—C5—C14^i^	123.17 (18)	C5J—C5K—C5L	119.8 (2)
C6—C5—C5A	118.53 (18)	C5J—C5K—H5KA	120.1
C14^i^—C5—C5A	118.30 (18)	C5L—C5K—H5KA	120.1
C5—C6—N21	126.01 (18)	C5M—C5L—C5K	120.3 (2)
C5—C6—C7	123.21 (18)	C5M—C5L—H5LA	119.8
N21—C6—C7	110.77 (18)	C5K—C5L—H5LA	119.8
C8—C7—C6	107.78 (19)	C5L—C5M—C5N	122.0 (2)
C8—C7—H7A	126.1	C5L—C5M—H5MA	119.0
C6—C7—H7A	126.1	C5N—C5M—H5MA	119.0
C7—C8—C9	106.63 (19)	C5A—C5N—C5M	122.31 (19)
C7—C8—H8A	126.7	C5A—C5N—C5I	119.88 (18)
C9—C8—H8A	126.7	C5M—C5N—C5I	117.81 (18)
N21—C9—C10	125.85 (19)	C10—C10A—C10B	112.21 (17)
N21—C9—C8	110.55 (17)	C10—C10A—H10A	109.2
C10—C9—C8	123.6 (2)	C10B—C10A—H10A	109.2
C9—C10—C11	121.1 (2)	C10—C10A—H10B	109.2
C9—C10—C10A	121.00 (19)	C10B—C10A—H10B	109.2
C11—C10—C10A	117.87 (18)	H10A—C10A—H10B	107.9
N22—C11—C10	126.47 (18)	C10C—C10B—C10A	113.98 (17)
N22—C11—C12	110.47 (18)	C10C—C10B—H10C	108.8
C10—C11—C12	123.1 (2)	C10A—C10B—H10C	108.8
C13—C12—C11	107.1 (2)	C10C—C10B—H10D	108.8
C13—C12—H12A	126.4	C10A—C10B—H10D	108.8
C11—C12—H12A	126.4	H10C—C10B—H10D	107.7
C12—C13—C14	107.17 (19)	C10B—C10C—C10D	113.40 (17)
C12—C13—H13A	126.4	C10B—C10C—H10E	108.9
C14—C13—H13A	126.4	C10D—C10C—H10E	108.9
N22—C14—C5^i^	125.85 (19)	C10B—C10C—H10F	108.9
N22—C14—C13	111.04 (18)	C10D—C10C—H10F	108.9
C5^i^—C14—C13	123.11 (18)	H10E—C10C—H10F	107.7
C5B—C5A—C5N	120.05 (18)	C10E—C10D—C10C	112.63 (18)
C5B—C5A—C5	120.47 (18)	C10E—C10D—H10G	109.1
C5N—C5A—C5	119.47 (17)	C10C—C10D—H10G	109.1
C5A—C5B—C5C	122.32 (19)	C10E—C10D—H10H	109.1
C5A—C5B—C5G	119.53 (19)	C10C—C10D—H10H	109.1
C5C—C5B—C5G	118.13 (18)	H10G—C10D—H10H	107.8
C5D—C5C—C5B	121.2 (2)	C10D—C10E—C10F	113.70 (18)
C5D—C5C—H5CA	119.4	C10D—C10E—H10I	108.8
C5B—C5C—H5CA	119.4	C10F—C10E—H10I	108.8
C5C—C5D—C5E	120.5 (2)	C10D—C10E—H10J	108.8
C5C—C5D—H5DA	119.8	C10F—C10E—H10J	108.8
C5E—C5D—H5DA	119.8	H10I—C10E—H10J	107.7
C5F—C5E—C5D	120.3 (2)	C10E—C10F—H10K	109.5
C5F—C5E—H5EA	119.9	C10E—C10F—H10L	109.5
C5D—C5E—H5EA	119.9	H10K—C10F—H10L	109.5
C5E—C5F—C5G	121.6 (2)	C10E—C10F—H10M	109.5
C5E—C5F—H5FA	119.2	H10K—C10F—H10M	109.5
C5G—C5F—H5FA	119.2	H10L—C10F—H10M	109.5

Symmetry codes: (i) −*x*, −*y*+1, −*z*.

## References

[bb1] Davis, N. K. S., Nicola, K. S., Pawlicki, M. & Anderson, H. L. (2008). *Org. Lett.* **10**, 3945–3947. 10.1021/ol801500b 18722461

[bb2] Fleischer, E. B., Miller, C. K. & Webb, L. E. (1964). *J. Am. Chem. Soc.* **86**, 2342–2348.

[bb3] Gallucci, J. C., Swepston, P. N. & Ibers, J. A. (1982). *Acta Cryst.* B**38**, 2134–2139.

[bb4] Hoard, J. L. (1973). *Ann. N. Y. Acad. Sci.* **206**, 18–31. 10.1111/j.1749-6632.1973.tb43202.x 4518386

[bb5] Hope, H. (1994). *Prog. Inorg. Chem.* **41**, 1–19.

[bb6] Lee, Y. J. & Scheidt, W. R. (1987). *Struct. Bonding (Berlin)*, **64**, 1–69.

[bb7] Rigaku (2008). *CrystalClear* . Rigaku/MSC, The Woodlands, Texas, USA.

[bb8] Runge, S., Senge, M. O. & Ruhlandt-Senge, K. (1999). *Z. Naturforsch. Teil B* **54**, 662–666.

[bb9] Senge, M. O. (2006). *Chem. Commun.* pp. 243–256. 10.1039/b511389j 16391725

[bb10] Senge, M. O., Bischoff, I., Nelson, N. Y. & Smith, K. M. (1999). *J. Porphyrins Phthalocyanines*, **3**, 99–116.

[bb11] Senge, M. O., Renner, M. W., Kalisch, W. W. & Fajer, J. (2000). *J. Chem. Soc. Dalton Trans.* pp. 381–385.

[bb12] Senge, M. O., Shaker, Y. M., Pintea, M., Ryppa, C., Hatscher, S. S., Ryan, A. & Sergeeva, Y. (2010). *Eur. J. Org. Chem.* pp. 237–258.

[bb13] Sheldrick, G. M. (2008). *Acta Cryst.* A**64**, 112–122. 10.1107/S0108767307043930 18156677

[bb14] Sooambar, C., Troiani, V., Bruno, C., Marcaccio, M., Paolucci, F., Listorti, A., Belbakra, A., Armaroli, N., Magistrato, A., De Zorzi, R., Geremia, S. & Bonifazi, D. (2009). *Org. Biomol. Chem.* **7**, 2402–2413. 10.1039/b820210a 19462051

[bb15] Volz, H. & Schäffer, H. (1985). *Chem. Ztg*, **109**, 308–309.

